# Testosterone-Induced Effects on Lipids and Inflammation

**DOI:** 10.1155/2013/183041

**Published:** 2013-03-31

**Authors:** Stella Vodo, Nicoletta Bechi, Anna Petroni, Carolina Muscoli, Anna Maria Aloisi

**Affiliations:** ^1^Department of Medicine, Surgery and Neuroscience, University of Siena, Via Aldo Moro 2, 53100 Siena, Italy; ^2^Department of Pharmacological and Biomolecular Sciences, University of Milan, Via Balzaretti 9, 20133 Milan, Italy; ^3^Department of Health Science, University of “Magna Graecia” Catanzaro and Drug Center, IRCCS San Raffaele Pisana, Via di Val Cannuta 247, 00163 Roma, Italy

## Abstract

Chronic pain has to be considered in all respects a debilitating disease and 10–20% of the world's adult population is affected by this disease. In the most general terms, pain is symptomatic of some form of dysfunction and (often) the resulting inflammatory processes in the body. In the study of pain, great attention has been paid to the possible involvement of gonadal hormones, especially in recent years. In particular, testosterone, the main androgen, is thought to play a beneficial, protective role in the body. Other important elements to be related to pain, inflammation, and hormones are lipids, heterogenic molecules whose altered metabolism is often accompanied by the release of interleukins, and lipid-derived proinflammatory mediators. Here we report data on interactions often not considered in chronic pain mechanisms.

## 1. Introduction

Chronic pain and inflammation involve multiple pathophysiological systems described or only suggested to be involved in their modulation, from genetic to environmental/cultural influences. Among all these actors, gonadal hormones have repeatedly been suggested to play a prominent role. Indeed, a number of studies have shown the ability of gonadal hormones to affect pain intensity and occurrence, for example [1]. Also important is the ability of pain (and pain therapies) to affect gonadal hormone metabolism, as recently reported by our group [2–4]. Patients often suffer complex side effects (fatigue, depression, osteoporosis, etc.) attributed to the original disease and not to the drug-induced endocrinopathies, and thus not adequately treated.

Gonadal hormones, androgen, and estrogen in particular are steroids present in both male and female subjects at different concentrations (Table 1), which depend mainly on age but are also highly sensitive to many internal and external factors. In both sexes, androgens are primarily synthesized in the gonads but also by the reticular portion of the adrenal gland as dehydroepiandrosterone (DHEA). The amount of testosterone (T) synthesized is regulated by the hypothalamic-pituitary-gonadal axis [5]. In males, T is reduced to 5*α*-dihydrotestosterone (DHT) by 5*α*-reductase (about 7%), an enzyme highly expressed in the urogenital tract, hair follicles, skin, liver, and brain [6]. In addition, 0.3% of T is converted to estradiol (E2) [7] by the enzyme aromatase, a member of the cytochrome P450 superfamily expressed in brain, liver, and adipose tissue. Testosterone and DHT bind to androgen receptors (AR) mostly located in the brain, skin, muscle, kidney, liver, and bone [8]. E2 is the most potent estrogen and targets a variety of tissues in the reproductive tracts, mammary gland and skeletal and cardiovascular systems. E2 acts by binding to its specific receptors (ER *α* and *β*). 

In the “classic” pathway of action, steroid hormones bind to their specific ligands and interact through the DNA binding domain with specific DNA sequences, activating or repressing transcription of target genes [9]. In addition to these well-known genomic effects of gonadal hormones, rapid effects appearing between seconds to a few minutes from stimulation have been described in different cell models [10]. 

Among the many effects of androgens and estrogens on body functions, we have concentrated on that between T and lipids, particularly in view of their involvement in inflammation and pain. Firstly, T is described as being involved in lipid modulation of inflammatory processes. Secondly, since obesity and other pathological or physiological conditions like aging can be accompanied by a hypogonadic state, we report data on the possible role played by this condition in the development of inflammation and pain. 

## 2. Lipids and Testosterone

The first step to be considered is the possible interactions between T and the other steroids, starting with cholesterol, its precursor. Cholesterol is the major constituent of cell membranes and serves as a precursor of important hormones and other substances. Cholesterol is insoluble in blood and is transported in the circulatory system bound to different lipoproteins. Low-density lipoproteins (LDL-C) carry cholesterol from the liver to cells of the body, particularly to organs that require it in large amounts (such as endocrine glands synthesizing steroids). The denser but smaller high-density lipoproteins (HDL-C), mainly consisting of lipoproteins and only a small cholesterol fraction, collect cholesterol from peripheral tissue and take it to the liver where it is metabolized [11]. It has been suggested that HDL-C and their protein and lipid constituents participate in body functions related to oxidation, inflammation, coagulation, and platelet aggregation [12]. 

The different concentrations of gonadal hormones in men and women are thought to be important factors contributing to the sex difference in lipoprotein profiles [13]. Epidemiological data suggest that T levels are negatively associated with total cholesterol, LDL-C, and triglyceride (TG) [14], while in men T levels appear to have a complicated and controversial relationship with HDL-C levels and cardiovascular risk. In fact, androgen levels within the normal adult male range were found to have a suppressive effect on HDL-C [15]. On the other hand, several studies on patients with coronary artery disease have shown that higher T levels are associated with higher HDL-C concentrations [16]. In particular, it was found that two genes involved in the catabolism of HDL-C are upregulated by T, namely, hepatic lipase (HL) and scavenger receptor B1 (SR-B1). SR-B1 mediates the selective uptake of HDL-C lipids into hepatocytes and steroidogenic cells, including Sertoli and Leydig cells of the testes, as well as cholesterol efflux from peripheral cells [5]. T upregulates SR-B1 in the human hepatocyte and in macrophages and thereby stimulates selective cholesterol uptake and cholesterol efflux, respectively. HL hydrolyzes phospholipids on the surface of HDL-C, facilitating the selective uptake of HDL-C lipids by SR-B1. The activity of HL is increased after administration of exogenous T [17]. The increases in both SR-B1 and HL activities are consistent with the total cholesterol lowering effect of T [5].

Obesity, and particularly visceral fat excess, is associated with insulin resistance, hyperglycemia, atherogenic dyslipidemia, and hypertension, as well as prothrombotic and pro-inflammatory states. Adiposity, with its associated hyperinsulinism, suppresses sex hormone-binding globulin (SHBG) synthesis and therewith the levels of circulating total T [18]. It may also decrease the strength of luteinizing hormone (LH) signaling to the testis [19]. In addition, insulin and leptin have a suppressive effect on testicular steroidogenesis [20, 21]. Visceral fat cells secrete a large number of cytokines which impair testicular steroidogenesis [22]. Hence there are reasons to believe that adiposity is a significant factor in lowering circulating levels of T. Furthermore, white adipose tissue, found in high levels in obese men, exhibits elevated aromatase activity and secretes adipose-derived hormones as well as adipokines. High levels of estrogens in obese males result from the increased conversion of androgens to estrogens, owing to the high bioavailability of these aromatase enzymes [23]. Hammoud et al. [24] recently discovered that an aromatase polymorphism modulates the relationship between weight and E2 levels in obese men. Abdominal or visceral fat is more likely to lead to changes in hormone levels and to cause inflammation than fat stored in other parts of the body [25]. An increase in aromatase activity also causes an alteration in the estrogen/T ratio, which may contribute to decreased androgen production.

Aromatase inhibitors were found to be an effective treatment in restoring normal hormone levels: this led to normalization of the patient's T, LH and FSH hormone levels, as well as suppression of the serum E2 levels [26]. 

## 3. Inflammation and Testosterone 

Inflammation is the body's response to cellular injury. The inflammation process involves several reciprocally modulating actors, from chemical factors derived from plasma proteins to cells that mediate vascular and cellular inflammatory reactions. To appreciate the inflammatory process, it is important to understand the role of chemical mediators such as eicosanoids, kinins, complement proteins, histamine, monokines, and cytokines, a group of soluble polypeptides. Even excess body fat can produce inflammation [27]. These inflammatory mediators act synergistically in the development of pain and hyperalgesia [28–30]. Cytokines are polypeptides produced by cells of both the innate and specific compartments of the immune system. There are various types of cytokines with widespread actions in the body. Many of these cytokines are produced by leukocytes, on which they also exert their key actions; it is common to call them interleukins (IL followed by a number). Although each one has a specific function, it is possible to identify common basic features: short period and self-limiting secretion, molecular weight between 10 and 50 kD, pleiotropic and redundant actions, influence on other cytokines (synthesis; action), systemic and local action, binding to membrane cell receptors [31]. These substances are known to be involved in changes to vascular permeability, the oxidative burst, and chemotaxis of leukocytes. 

In some cases, especially in the elderly, the body loses its ability to stop the cytokine secretion [32]; indeed, aging is accompanied by a pro-inflammatory state expressed by the increasing levels of several cytokines, including interleukin-6 (IL-6). The need to focus attention on aging derives from the evidence that in men over 45–50 years there is a progressive, slow, but continuous decrease of serum T levels, and androgens have been shown to inhibit the expression and release of cytokines and chemokines [33, 34]. This relationship is supported by the finding that androgen deprivation therapy is associated with increased levels of pro-inflammatory factors and decreased levels of anti-inflammatory cytokines [35, 36], while observational and interventional studies indicate that T supplementation reduces inflammatory markers in both young and old hypogonadal men [35]. 

Moreover, several lines of evidence support a close association between T levels, the evolution of diabetes secondary to hyperglycemia and hyperlipidemia and oxidative stress [37]. This association is most likely the result of elevated metabolic rates required to maintain normal biological processes and an increased level of stress in the local testicular environment, both of which naturally produce reactive oxygen species (ROS). 

As ROS are generated mainly as by-products of mitochondrial respiration, mitochondria are thought to be the primary target of oxidative damage and play an important role in aging. Emerging evidence has linked mitochondrial dysfunction to a variety of age-related diseases, including neurodegenerative diseases, cancer, and chronic inflammation [38].

Oxidative stress is the result of an imbalance between the production of ROS and antioxidant defenses [39, 40]. In particular, ROS and reactive nitrogen species (RNS) are unstable and very reactive by-products of normal metabolism, leading to lipid peroxidation, nucleic acid oxidation (including DNA modification and DNA strand breaks), protein oxidation, and enzyme inactivation [39, 41–43]. 

Lipid peroxidation refers to the addition of oxygen to unsaturated fatty acids to form organic hydroperoxides (ROOH). Organic peroxyl (ROO^*∙*^) radicals arise during the radical-initiated and O_2_-dependent peroxidation of lipids, which can also produce alkoxyl radicals (RO^*∙*^) in metal-catalyzed reactions [44]. The oxidation of membrane phospholipids in the plasma membrane, as well as within internal organelle membranes such as the mitochondria, leads to biophysical changes that disrupt membrane and organelle function. While these processes may stimulate cellular signaling pathways, they are generally associated with the promotion of cell death. Breakdown of lipid peroxidation yields additional reactive species (e.g., 4-hydroxynonenal, 4-HNE and malonyldialdehyde), which may contribute to toxicity and/or cellular signaling [45]. In addition, an increase in lipid peroxidation may be one of the factors responsible for the disruption of the normal feedback mechanism in the hypothalamus-pituitary-gonadal (HPG) axis [46]. 

Since T usually enhances the metabolic rate [47, 48], it could be expected that high T levels might alter the balance between ROS production and antioxidant defenses, resulting in an enhanced risk of oxidative stress [49, 50]. Yet, closer scrutiny of the available data reveals a more complex pattern, and different studies indicate that the relationship between T and oxidative stress can be more complex than previously thought, as it is tissue- and gender-dependent [51, 52]. 

## 4. Testosterone, Aging, and Inflammation

Aging is associated with a decrease in circulating T levels. This characteristic hormonal change of male aging is of interest because lower T concentrations are commonly associated with a number of clinical conditions of particular importance such as metabolic syndrome, type 2 diabetes, carotid intima-media thickness, and aortic and lower limb arterial disease [53–55]. The effects related to the cardiovascular system are particularly important because of the high personal and economic costs. Putative mechanisms by which lower T levels could contribute to an increased burden of cardiovascular disease range from the loss of beneficial effects of T on endothelial function and vasodilation to epidemiological correlations between T and more favorable lipid profiles [56, 57]. Indeed, lower T is associated with higher body mass index and fat mass, which are recognized cardiovascular risk factors. A study by Nettleship et al. [58] provided evidence that low serum T is linked to increased fatty streak formation. Moreover, as already reported, many of these conditions present in the elderly are accompanied by a pro-inflammatory state expressed by the increasing levels of inflammatory cytokines, including interleukin-6 (IL-6), tumor necrosis factor alpha (TNF-alpha), and interleukin-1 beta (IL-1beta). 

These inflammatory cytokines are known to modulate lipid homeostasis, vascular endothelial function, plaque, and atherosclerosis. During inflammation, peroxynitrite, a potent pro-inflammatory nitro-oxidative species with an established role in inflammation [59], induces endothelial cell damage and increased microvascular permeability [60] and activates redox-sensitive transcription factors, including NF-*κ*B and AP-1, which in turn regulate genes encoding the pro-inflammatory and pronociceptive cytokines such as IL-1*β*, TNF-*α*, and IL-6 [61, 62]. Peroxynitrite also upregulates adhesion molecules such as ICAM-1 and P-selectin to recruit neutrophils at sites of inflammation [63] and autocatalyzes the destruction of neurotransmitters and hormones such as norepinephrine and epinephrine [64]. Age-associated induction of NF-*κ*B activation is especially interesting since it seems to contribute significantly to endothelial activation in aged vessels, a critical initial step in the development of atherogenesis [65]. A significant clinical example of the possible interaction between these factors is peripheral artery disease (PAD), consisting of partial or complete obstruction of the arteries in the lower limbs; it is one of the most common manifestations of atherosclerosis and is more frequent in aging men. Patients often describe claudication pain as episodic, which may be accompanied by physical findings of foot blanching and disappearance of pedal pulses. This was attributed primarily to a flow-limiting stenosis or occlusion of a conduit artery that limits oxygen delivery during exercise. A large body of evidence indicates that, with exercise, limb ischemia evokes an acute systemic response characterized by increased oxidative stress, local and systemic inflammation and endothelial dysfunction [66, 67]. In patients with claudication, these inflammatory responses to exercise may have adverse interactions with both the microcirculation and skeletal muscle metabolism, which could further compromise exercise performance and increase pain. 

## 5. Vitamin D, Testosterone, and Inflammation 

Vitamin D, in particular its metabolite 25-hydroxyvitamin D (25[OH]D), is widely recognized for its involvement in calcium homeostasis and immunomodulatory effects. Its hormonal action decreases the risk of many chronic illnesses, including osteoporosis, osteoarthritis, metabolic syndrome, fibromyalgia, and chronic fatigue syndrome [68–70]. Vitamin D can be synthesized in the skin from sun exposure and is found in salmon, mushrooms, eggs, and dairy products.Biological actions of vitamin D are mediated through the vitamin D receptor (VDR). The VDR is almost ubiquitously expressed in human cells, which underlines the clinical significance of the vitamin D endocrine system [68]. Altered vitamin D homeostasis is associated with increased risk of developing obesity [71, 72], hypertension [73], glucose intolerance, and metabolic syndrome [74]. Indeed, plasma vitamin D levels were associated inversely with body mass index (BMI) and fat levels and positively with HDL cholesterol [75]. Furthermore, visceral adipose tissue was higher in vitamin D deficient subjects. Sequestration of vitamin D in body fat stores and its consequent reduced bioavailability offer a plausible explanation for this association [76, 77]. Recent research revealed that calcitriol also exhibits multiple anti-inflammatory effects. First, calcitriol inhibits the synthesis and biological actions of pro-inflammatory prostaglandins (PGs) by three mechanisms: suppression of the expression of cyclooxygenase-2, the enzyme that synthesizes PGs; upregulation of the expression of 15-hydroxyprostaglandin dehydrogenase, the enzyme that inactivates PGs; and downregulation of the expression of PG receptors that are essential for PG signaling [78]. Moreover, vitamin D is able to suppress the release of TNF-*α* and to enhance synthesis of the anti-inflammatory cytokine IL-10 [79, 80]. Finally, vitamin D enhances the effect of anti-estrogen-like substances. In addition to these general/indirect effects, it has been shown that vitamin D increases T levels. This is primarily due to vitamin D being able to decrease the enzyme aromatase, which converts T into E2. 

In fact, vitamin D reduces the production of E2 itself and blocks the production of the alpha-E2 receptor [81]. Thus, vitamin D increases T levels, as further confirmed by a study in which men with sufficient 25(OH)D levels had significantly higher levels of T and significantly lower levels of SHBG than 25(OH)D-insufficient men [82]. Moreover, Pilz and colleagues reported that vitamin D supplementation increases T levels [83]. Symptoms of T deficiency, which may be indirectly contributed to by a lack of vitamin D, include fatigue, depression, and muscle wasting. This reduced muscle mass could promote pain in muscles, causing older men to attribute muscle aches and pains to the aging process.

## 6. Clinical Aspects

As we have shown, there are various problems related to androgen dysfunction and inflammation such as fatigue, obesity, glycemic imbalance and altered immunity. These may represent the precursors of more severe conditions leading to disease in many individuals [84–86]. 

The neurodegenerative disorder X-linked-adrenoleukodystrophy (X-ALD) is an example of interesting links between T, lipid metabolism and inflammation. In X-ALD, a certain percentage of patients present hypogonadism. Moreover, due to the mutation of a peroxisomal transport protein, the metabolic pathways of specific long chain fatty acids (FA, very long chain fatty acids) are impaired [87, 88]. These FA accumulate abnormally in plasma and in all tissues, although the most affected ones are the nervous system, the adrenal and the testis, all characterized by elevated steroidogenesis. FA can be esterified in different forms, an important component being FA esterified with cholesterol. They are vehicled by lipoproteins. The adrenal cortex and testis of affected patients contain intracytoplasmic lamellar inclusions consisting of FA-cholesteryl esters [89]. Cholesterol, as mentioned above, can be metabolized into androgens. In steroidogenic tissues, free cholesterol can be obtained in three ways: after cholesteryl ester hydrolysis, de novo synthesis from acetate, or mainly imported from lipoproteins by specific receptor-mediated pathways. In the adrenals, this mechanism is mediated by adrenocorticotropic hormone (ACTH).

In X-ALD, since cholesterol is entrapped as esters in the lamellar inclusions, it cannot be normally metabolized into T. Moreover, the functionality of the T-converting enzyme 5*α*-reductase is altered in X-ALD [90, 91], indicating an alteration of the homeostasis of androgens. In X-ALD and in other chronic disorders, alterations of lipid metabolism, such as FA peroxisomal catabolism and esterification processes, and the presence of secondary inflammation, augmented by the release of interleukins and lipid-derived pro-inflammatory mediators, can contribute to a T deficit or generally to an alteration of T homeostasis and to the consequent clinical symptoms of the patients.

## 7. Conclusion (See Figure 1)

Androgens are large functional molecules able to greatly affect body functions. In this paper, we have considered the relationships between the main androgen hormone, T, and some aspects of inflammatory processes in order to highlight possible mechanisms able to affect pain chronicization. Indeed, it is becoming increasingly clear that inflammation, often not clearly acknowledged, is involved in many chronic painful syndromes still far from being explained by the “usual” pain system alterations. 

## Figures and Tables

**Figure 1:**
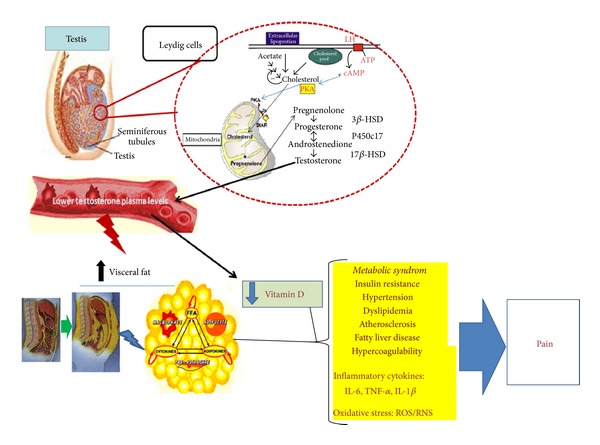
Representative schema of the clinical consequences suggested to be related to androgen deficiency. Lower testosterone levels are associated with an increased metabolic risk, systemic inflammation, and chronic pain.

**Table 1:** Hormone levels commonly recorded in adult men and women. In females, the high variability of estradiol concentration is due to the menstrual cycle variations. Note that testosterone is expressed in ng/mL and estradiol in pg/mL (1 ng = 1000 pg).

Hormones	Adult men	Adult women
Testosterone (ng/mL)	3–8	0.5–1
Estradiol (pg/mL)	<50	20–400
Estriol (mg/dL)	<2	<2
Estrone (pg/mL)	15–65	Pre-menopausal: 15–200
		Post-menopausal: 15–55
Androstenedione ng/dL	50–220	30–285
SHBG nmol/L	14–71	20–155
DHEA ng/dL	180–1250	130–980
DHEA-Sulfate *µ*g/dL	10–619	Pre-menopausal: 12–535
		Post-menopausal: 30–260
